# Development of long-acting riluzole transdermal patch against amyotrophic lateral sclerosis: Mechanistic insights into polyglyceryl-3 dioleate-enhanced drug release and skin permeation

**DOI:** 10.1016/j.ijpx.2025.100363

**Published:** 2025-07-20

**Authors:** Yanan Liu, Guixue Chen, Maojian Li, Man Li, Daoxuan Xie, Zheng Luo

**Affiliations:** Department of Pharmaceutical Sciences, College of Pharmacy, Beihua University, Jilin 132013, PR China

**Keywords:** Riluzole, Long-acting transdermal patch, Chemical penetration enhancer, Transdermal absorption, Molecular interactions

## Abstract

Patients with amyotrophic lateral sclerosis (ALS) often experience difficulty swallowing, making oral administration unsuitable for effective treatment. A transdermal drug delivery system (TDDS) offers a long-acting, non-invasive alternative for ALS therapy. In this study, a riluzole transdermal patch capable of sustained release over 72 h was developed. In vitro skin permeation and pharmacokinetic experiments were conducted to evaluate the impact of various factors—including drug loading, type and concentration of chemical penetration enhancers (CPEs), and type of pressure-sensitive adhesive—on riluzole absorption through the skin. The optimized patch formulation contained 17 % (*w*/w) riluzole and 10 % (w/w) polyglyceryl-3 dioleate (PGD), with an adhesive layer thickness of 111 μm. The final prescription penetration rate of riluzole was found to be 2.96 μg/(h·cm^2^). Optimized formulation displayed enhanced stability and prolonged pharmacokinetic performance (*C*_max_ = 74.34 ± 13.62 ng/mL, MRT_0-*t*_ = 34.91 ± 11.31 h). No significant skin irritation was observed. The role of PGD in the in vitro release and in vivo transdermal absorption of riluzole was thoroughly investigated. The results revealed that PGD not only reduced the interaction between riluzole and the pressure-sensitive adhesive, enhancing drug release but also increased the fluidity of skin lipids, leading to improved transdermal absorption. This study provides a comprehensive molecular-level understanding of PGD's effect on riluzole permeation, offering valuable insights for the rational selection of CPEs in the development of riluzole TDDS.

## Highlights

• A novel long-acting riluzole transdermal patch was developed.
• Polyglyceryl-3 dioleate enhanced riluzole release from transdermal patch.
• Polyglyceryl-3 dioleate increased the fluidity of skin lipids.

## Graphical abstract

**Figure:**
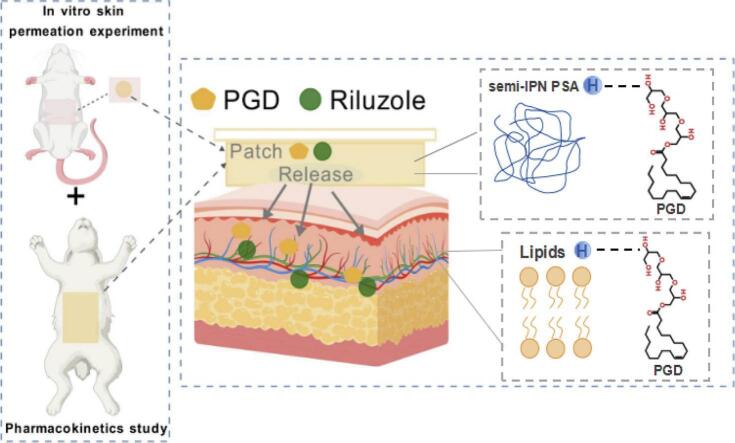

## 1 Introduction

Riluzole, a benzothiazole-based compound and glutamate release inhibitor, is the first drug approved by the U.S. Food and Drug Administration (FDA) for the treatment of Lou Gehrig's disease clinically referred to as amyotrophic lateral sclerosis (ALS). Riluzole treatment has been demonstrated to slow down disease progression and prolong survival in patients with ALS (Andrews et al., 2020; Salminen et al., 2021). The therapeutic action of riluzole stems from its ability to inhibit glutamate release, block voltage-gated sodium channels, and modulate neurotransmitter signaling, collectively reducing excitotoxic neuronal damage (Bellingham, 2011). However, oral administration of riluzole is frequently accompanied by fatigue, nausea, dizziness, and hepatic dysfunction. Dysphagia—a common symptom in patients with ALS (Brooks et al., 2019), further impairs oral medication adherence, restricting overall clinical effectiveness (Teixeira et al., 2023).

**Fig. 1:**
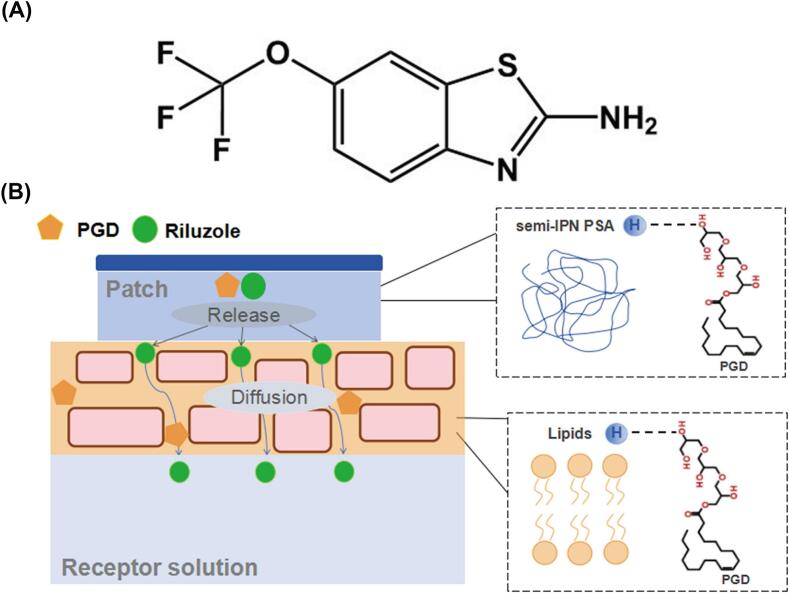
(A) Chemical structure of riluzole. (B) Schematic diagram of riluzole release from patch and penetration into skin tissue.

Transdermal drug delivery systems (TDDSs) have attracted considerable interest as a non-invasive alternative for various diseases. By surpassing the first-pass hepatic metabolism, and maintaining sustained plasma drug levels TDDS considerably improves patient compliance. (Bednarczyk et al., 2023). TDDS delivers drugs through the skin (Alkilani et al., 2015), rendering them particularly suitable for the long-term management of chronic conditions. In response to the challenges associated with ALS treatment, there is an increasing need for the development of a long-acting riluzole transdermal patch. As illustrated in Fig. 1A, the chemical structure of riluzole, along with its low daily dosage of 100 mg, positions it as an ideal candidate for TDDS. Riluzole exhibits a log *P* 2.3 and water solubility 0.0395 mg/mL. Based on the classification criteria where compounds with water solubility <0.1 mg/mL are considered poorly soluble and those with log *P* > 2.0 demonstrate high permeability, riluzole is classified as a Biopharmaceutics Classification System (BCS) Class II drug, characterized by low solubility and high permeability. Its physicochemical characteristics—molecular weight of 234.20 Da, a melting point ranging from 116 to 118 °C, log *P* of 2.3—further enhance its suitability for transdermal delivery (Liu et al., 2016; Sun et al., 2023). The creation of sustained-release transdermal patches for riluzole has the potential to significantly decrease dosing frequency, while maintaining consistent plasma concentrations through controlled drug release, therefore offering a novel approach to ALS treatment. This would optimize pharmacokinetic profiles and improve patient compliance.

Chemical penetration enhancers (CPEs) are a well-established method for modulating drug permeation rates in TDDS. These enhancers primarily function by disrupting the lipid bilayer structure of the stratum corneum (SC), thus increasing skin permeability. CPEs can be categorized into six main groups: surfactants (e.g., lecithin, sodium dodecyl sulfate), terpenes (e.g., menthol, limonene), cell-penetrating peptides, organic solvents (e.g., ethanol, dimethyl sulfoxide), fatty acids and esters (e.g., oleic acid, isopropyl myristate), and other compounds (e.g., azones, cyclodextrins). Surfactant-based CPEs represent commonly used CPEs in TDDSs (Shivani, 2021). This dual affinity enables them to interact effectively with the highly ordered lipid matrix of the SC, facilitating its disruption. The enhancement of transdermal drug delivery by surfactants is primarily achieved through three interconnected mechanisms. One key mechanism involves the insertion of surfactant molecules into the SC lipid bilayers, leading to disruption of their structural organization and an increase in lipid fluidity within the intercellular spaces. Surfactants also bring about reversible conformational changes in skin proteins by disrupting the hydrogen-bonding network within corneocyte keratin. Spectroscopic studies have shown that SDS, in particular, alters the secondary structure of keratin, facilitating the transient formation of aqueous pores. These structural modifications temporarily increase the permeability of the SC barrier. Micellar solubilization has also been observed to play a significant role in enhancing drug permeation. Above their critical micelle concentration (CMC), surfactant monomers spontaneously self-assemble into micelles, capable of encapsulating hydrophobic drug molecules. This encapsulation improves the solubility of poorly water-soluble compounds and facilitates their partitioning into the lipophilic SC. Lipid matrix disruption, protein conformational modulation, and micellar solubilization thus represent the multifaceted mechanisms by which surfactants facilitate transdermal drug delivery. Lecithin-based micelles have been shown to significantly improve the transdermal transport of ceramide AP (Sahle et al., 2013). Surfactant-based CPEs are widely employed in TDDS and a comprehensive understanding of their permeation enhancement mechanisms is essential for the informed and strategic selection of CPEs in transdermal formulations.

In TDDS, drug delivery involves two pivotal stages: the release of the drug from the patch matrix and its subsequent permeation through the skin barrier (Li et al., 2017). Traditional viewpoints assert that the primary function of the CPEs is to enhance drug permeation by altering the microstructural organization of the SC (Rachakonda et al., 2008). The relatively new concept of “release-permeation synergistic enhancement” highlights the multidimensional regulatory potential of CPEs in TDDS; not only do they facilitate drug permeation through modulation of the SC, but also influence drug release by precise regulation of diffusion kinetics. Achieving this involves controlled microstructural modifications of the polymeric matrix, such as optimizing free volume and modulating molecular interactions (Luo et al., 2021; Song et al., 2016), —alongside barrier modulation, including SC lipid fluidization and conformational changes in skin proteins (Zhang et al., 2015).

This study focused on the development of a riluzole transdermal patch with a sustained release over 72 h, as a non-invasive transdermal route of administration for riluzole, and to elucidate the precise mechanism by which CPEs enhance its transdermal delivery. Polyglyceryl-3 dioleate (PGD), a surfactant-based CPE, demonstrated a pronounced ability to promote the transdermal absorption of riluzole. The underlying enhancement mechanism is explored from two key perspectives, corresponding to the two critical stages of the drug permeation process: release from the patch matrix and absorption across the skin. As illustrated in Fig. 1B, drug release is primarily governed by the physicochemical characteristics of the patch formulation, whereas transdermal absorption is largely determined by the barrier properties of the SC, which can be effectively modulated by CPEs. Moreover, the adhesive performance of the pressure-sensitive adhesive layer plays a vital role in ensuring therapeutic efficacy (Wang et al., 2025). A novel semi-interpenetrating polymer network pressure-sensitive adhesive (semi-IPN PSA) was therefore applied to increase the adhesive property of TDDS, as reported by our previous study (Chen et al., 2025). In vitro transdermal experiments were conducted to evaluate drug loading, types and contents of CPEs, and PSA types. The Box-Behnken design (BBD) is a three-level response surface methodology particularly suited for investigating nonlinear effects of continuous variables (e.g., drug loading, permeation enhancer content, patch thickness) on responses (e.g., skin permeation). This approach is ideal for nonlinear analysis of continuous variables requiring balanced efficiency and precision, as demonstrated in previous studies (Song et al., 2022; Sun et al., 2023; Wu et al., 2024; Xu et al., 2024). This study employed the BBD to optimize the formulation of a riluzole transdermal patch by systematically investigating the synergistic effects of drug loading (*X*_1_: 10–20 %), permeation enhancer PGD content (*X*_2_: 5–15 %), and patch thickness (*X*_3_: 80–140 μm) on skin permeation amount (*Y*). A quadratic response surface model was established through 17 experimental runs (including 5 center points). Mechanistic studies on permeation enhancement and pharmacokinetic assessments were then carried out on the optimized patch formulation. The interactions among the drug, CPE, and semi-IPN PSA were investigated via differential scanning calorimetry (DSC), Fourier-transform infrared spectroscopy (FT-IR), ^13^C nuclear magnetic resonance (^13^C NMR), rheological studies, and molecular docking to probe into their thermodynamic and molecular-level interactions. The interaction between the drug, CPE, and skin was further examined using attenuated total reflectance FT-IR, confocal laser scanning microscopy (CLSM), Raman spectroscopy, small-angle X-ray scattering (SAXS), and molecular simulations.

## 2 Materials and methods

### 2.1 Materials

Riluzole, 2-hydroxyethyl acrylate (HEA), 2-ethylhexyl acrylate (EHA), acrylamide (AM), acrylic acid (AA), methyl acrylate (MA), isooctyl ester, 2-hydroxy-2-methylpropiophenone, and trimethylolpropane triacrylate were purchased from Aladdin Biochemical Technology Co., Shanghai, China. DURO-TAK® 87–2287 and DURO-TAK® 87–4098 were obtained from Henkel Corporation (Düsseldorf, Germany), while other PSAs were synthesized in our laboratory following a previously reported study (Chen et al., 2025). Scotchpak™ 9755 release liner and Scotchpak™ 9720 backing film were sourced from 3 M (St. Paul, MN, USA). Monosteol™, PGD, Isopropyl myristate, Laurocapram, Peceol™, Transcutol P, Capryol 90, and LABRAFIL M1944 CS were purchased from Gattefossé (Shanghai, China). All other chemicals were of reagent grade and commercially available.

Wistar rats (male, 200 ± 20 g) were obtained from Yisi Experimental Animal Technology Co., Ltd. (Changchun, China), while Japanese white rabbits (male, 2.0 ± 0.5 kg) were sourced from Liaoning Changsheng Biotechnology Co., Ltd. (Shenyang, China). All animal experiments were performed in compliance with the National Institutes of Health (NIH) Guide for the Care and Use of Laboratory Animals and received approval from the Animal Ethics Committee of Beihua University (Approval No. BHU-IACUC-C2022-4-20).

### 2.2 Optimization of formulation

#### 2.2.1 Preparation of riluzole patch

The pure semi-IPN PSA was synthesized via UV-induced photopolymerization using 2-hydroxy-2-methylpropiophenone (2 wt%) as the photoinitiator. A homogeneous solution was prepared by dissolving pre-synthesized linear PSA (LPSA) and trimethylolpropane triacrylate (9:1 mass ratio) in ethyl acetate under continuous stirring for 3 h. The mixture was then coated onto a Scotchpak™ 9755 release liner using a 0.45 mm applicator to form a film. Photocrosslinking was initiated by 365 nm UV irradiation (250 W) to form the semi-IPN architecture. The cured semi-IPN PSA was air-dried at ambient temperature for 15 min followed by oven-drying at 60 °C for 45 min to remove residual solvent. The linear PSA and poly(trimethylolpropane triacrylate) jointly constitute a semi-IPN, wherein the linear polymer component provides essential adhesive properties to the transdermal patch, while the covalently crosslinked network imparts superior mechanical strength to the transdermal patch (Li et al., 2024).

The solvent evaporation method was used to prepare the riluzole patch (Song et al., 2022). Riluzole (with or without CPEs) was first dissolved completely in ethyl acetate. The LPSA and trimethylolpropane triacrylate were then incorporated into the drug solution, and the mixture was stirred magnetically for 2 h at room temperature until fully dissol*v*ed. Following the addition of 2-hydroxy-2-methylpropiophenone, the solution was shielded from light and stirred for an additional 30 min, then allowed to stand for 10 min. The PSA solution was then applied to a release liner (Scotchpak™ 9755) using a 0.45 mm applicator to form a film. The film was exposed to ultraviolet light for 30 s, left to stand for 15 min at room temperature, and subsequently placed in an oven at 50 °C for 15 min to evaporate the solvent. Finally, the patch was removed, covered with a release liner or backing layer, and cut into the desired sizes for use.

#### 2.2.2 Preparation of isolated skin

The skin preparation was performed following methods reported in a previous study (Zhang et al., 2021a). The abdominal hair of the rats was carefully removed after anesthetizing the rats with urethane (20 %, *w*/*v*), using scissors and an electric razor. After euthanasia, the full-thickness abdominal skin was carefully excised and subcutaneous fat was removed. The skin samples were cleaned and the skin integrity was assessed microscopically, following which the specimens were stored at −80 °C, and used within 30 days.

#### 2.2.3 In vitro skin permeation of patch

An in vitro transdermal experiment of the riluzole patch was conducted using a horizontal Franz diffusion cell (Liu et al., 2019). The effective diffusion area measured 1.13 cm^2^. One side of the backing layer was carefully peeled off the circular riluzole patch and the patch was applied to the skin, ensuring the dermis was positioned towards the receptor compartment. The diffusion cell was then secured and positioned in a multifunctional transdermal diffusion apparatus. The receiving medium comprised a potassium dihydrogen phosphate‑sodium hydroxide aqueous solution (PBS, pH 7.4) with 0.1 % sodium azide (4 mL). The system was operated at a constant temperature of 32 °C, with the receptor compartment stirred at 600 rpm to ensure uniform mixing. At predetermined time points (2, 4, 6, 8, 10, 12, 24, 30, 36, 48, 60, and 72 h), 2 mL of the receptor medium was carefully drawn from the receptor compartment and immediately replaced with an equal volume of fresh medium to maintain sink conditions and prevent air bubble formation. The collected samples were centrifuged under refrigerated conditions, and the resulting supernatants were analyzed using HPLC. A plot of the cumulative drug permeation per unit area versus time was generated to assess the in vitro transdermal drug deli*v*ery profile.

The cumulative drug skin permeation amount was determined by the concentration and volume of each sampling point.

(1) $$Q=\left(\sum_{i=2}^{n}2.5C_{i}-0.5C_{i-2}\right)/A\;\left(i=2,46\right)$$

where *Q* was the cumulative skin permeation amount. *C*_i_ was the concentration in the recei*v*er compartment at time *i* and *A* was the effective diffusion area (1.12 cm^2^). The cumulative amount permeated from unit area versus time *i* was plotted (Liu et al., 2017).

#### 2.2.4 Quantitative Analysis of riluzole

Riluzole quantification was performed using a validated HPLC method. The HPLC system (LC-2010 A HT) was equipped with an L-2130 pump, an L-2200 autosampler, and an L-2420 UV absorbance detector. Chromatographic separation was achieved using an ODS C18 column (250 × 4.6 mm, 5 μm) maintained at 40 °C. For in vitro transdermal samples, the mobile phase consisted of methanol, water, triethylamine, and phosphoric acid in a ratio of 85:15:0.3:0.3 (*v*/v). Detection was carried out at 254 nm with a flow rate of 1 mL/min and an injection volume of 20 μL. For in vivo pharmacokinetic samples, the mobile phase comprised acetonitrile, methanol, and 0.1 M ammonium acetate in a ratio of 3:2:5 (v/v) (Sarkar et al., 2018). The injection volume was 50 μL, the detection was made at 263 nm, and the flow rate was maintained at 1 mL/min. All analytical procedures were thoroughly validated to ensure accuracy and reliability.

#### 2.2.5 Optimization of drug and excipient content

The patch formulation was first evaluated through single-factor experiments to identify influential variables. Subsequently, Design Expert 13 software was used to optimize the formulation components—specifically riluzole concentration, PGD content, and patch thickness—using a three-level BBD. The cumulative drug permeation through the skin was chosen as the response variable. The optimization objective was to maximize this response, with equal emphasis placed on all selected factors to determine the most effective formulation parameters. We chose 80–140 μm patch thickness for testing (Maillard-Salin et al., 2000; Song et al., 2022; Sun et al., 2023). Thick patches reduce drug release and skin permeation amount, causing a decrease in availability (Lv et al., 2016). Thin patches deliver less drug (Maillard-Salin et al., 2000). For CPE content, we selected 5–15 % CPE. The CPE content less than 5 % may be ineffective, while CPE amount more than 15 % may cause skin irritation (Ruan et al., 2022).

#### 2.2.6 Pharmacokinetics study

Japanese long-eared white rabbits (2.5 ± 0.1 kg) were randomly assigned to four groups (*n* = 6 per group): an intravenous injection group, an oral gavage group, a control group receiving a standard riluzole patch, and a group treated with the optimized riluzole patch. For the patch application, the abdominal hair of rabbits in different groups was shaved one day before the experiment. Throughout the study period, all animals were provided with unrestricted access to food and water.

For the intravenous group, riluzole was formulated as a 5 mg/mL solution, using a solvent mixture of PEG 400, propylene glycol, glycerin, and ultrapure water in a volume ratio of 3:4:2:11. The solution was administered via the marginal ear vein at a dose of 0.6 mL. Blood samples (approximately 0.4 mL) were collected from the ear vein at predetermined time points: 0.08, 0.25, 0.5, 1, 2, 3, 4, 6, 8, 10, 12, and 24 h post-administration. In the oral gavage group, the same 5 mg/mL riluzole solution and 0.6 mL dose was administered. Blood samples (approximately 0.4 mL) were collected from the ear vein at predetermined time points: 0.5, 1, 2, 3, 4, 5, 6, 7, 8, 10, 12, 24, and 48 h post-administration. To ensure consistent drug exposure, both the control group (standard riluzole patch) and the optimized patch group received transdermal patches measuring 5 × 10 cm^2^ (95 mg). After 72 h of patch application, the patches were removed, and blood samples were collected at designated time intervals: 1, 2, 4, 6, 8, 10, 12, 14, 24, 28, 32, 36, 48, 54, 60, 72, 78, 84, and 96 h. Plasma was separated by centrifugation at 16,000 rpm for 8 min at 4 °C, and the supernatant was stored at −80 °C until analysis.

Plasma samples containing riluzole were processed using a liquid-liquid extraction method using ethyl acetate as the extraction solvent. In brief, 100 μL of plasma was transferred into centrifuge tubes, followed by the addition of 10 μL of an internal standard solution (5-MOP, 10 μg/mL) and 1 mL of ethyl acetate. The mixture was vortexed for 3 min and then centrifuged at 16,000 rpm for 5 min. The resulting organic phase was carefully transferred to a clean tube and evaporated to dryness under a gentle stream of nitrogen at 45 °C. The dry residue was reconstituted in 100 μL of mobile phase, filtered through a 0.22 μm membrane, and subsequently analyzed using HPLC (Sarkar et al., 2018).

Riluzole and the internal standard 5-MOP were respectively eluted at retention times of approximately 9.0 min and 7.4 min. Over the riluzole concentration range of 0.78 to 500 ng/mL, the method demonstrated excellent linearity, with a correlation coefficient (r) of 0.9941. The lower limit of quantification (LLOQ) was established at 0.78 ng/mL. Pharmacokinetic parameters were calculated using DAS Version 3.0 based on compartmental models, utilizing experimentally determined plasma drug concentration-time data. Non-compartmental analysis (statistical moment theory) was employed to derive key parameters including AUC, *C*_max_, and *t*_1/2_, while dose proportionality was evaluated using a power model (Li et al., 2016; Sun et al., 2013). And calculate its relative bioavailability (*F*_re_) and absolute bioavailability (*F*_ab_) according to the formula (2–3).

(2) $$F_{\mathrm{re}}\left(\%\right)=\frac{{\mathrm{AUC}}_{\mathrm{Optimized riluzole patch}}\times {\mathrm{dose}}_{\mathrm{riluzole patch}}}{{\mathrm{AUC}}_{\mathrm{riluzole patch}}\times {\mathrm{dose}}_{\mathrm{Optimized riluzole patch}}}\times 100\%$$

(3) $$F_{\mathrm{ab}}\left(\%\right)=\frac{{\mathrm{AUC}}_{\mathrm{patch}}\times {\mathrm{dose}}_{i.v.}}{{\mathrm{AUC}}_{i.v.}\times {\mathrm{dose}}_{\mathrm{patch}}}\times 100\%$$

#### 2.2.7 Skin irritation experiment

A skin irritation test was conducted on the abdominal area of male Japanese long-eared white rabbits (Kandarova et al., 2018). Twelve hours before administration, the abdominal hair of the rabbits was carefully shaved to preserve skin integrity. The animals were then randomly assigned to five groups: a negative control group (no treatment), a positive control group (0.5 mL of 10 % SDS solution applied for 4 h), a blank patch group, a riluzole patch group, and an optimized riluzole patch group containing PDG. After 12 h application, the patches were taken off, and the application sites were cleansed with warm saline. Any signs of erythema or edema were recorded as photographs taken at 12 and 24 h after patch removal.

#### 2.2.8 H&E staining experiment

Histological analysis of the treated skin was conducted using hematoxylin and eosin (H&E) staining. After completing the skin irritation assessment, the rabbits were humanely euthanized. The tissues excised from application sites were fixed in 4 % paraformaldehyde, embedded in paraffin, and sectioned. These sections were then stained with H&E and examined microscopically to assess any structural changes or signs of irritation at the histological level (Li et al., 2024).

### 2.3 Mechanism of the Enhancement effect of PGD

#### 2.3.1 Drug release process

##### 2.3.1.1 In vitro drug release experiment

The in vitro release experiment was conducted using a horizontal single-chamber diffusion cell (Nan et al., 2022). A Cellophane® semipermeable membrane was employed as the release barrier. The riluzole patch, prepared in-house, was affixed to the membrane, and PBS (pH 7.4; 4 mL) served as the receptor medium. The experiment was conducted using a multifunctional transdermal diffusion apparatus maintained at 32 °C, with continuous stirring of the receptor phase at 600 rpm to ensure uniform distribution. At predetermined time points (0.5, 1, 2, 3, 4, 6, 8, 12, and 24 h), 2 mL samples were withdrawn from the receptor compartment and immediately replaced with an equal volume of fresh PBS to preserve sink conditions. The analysis of riluzole concentration was performed using HPLC, following the same method described for the in vitro transdermal permeation studies.

##### 2.3.1.2 FT-IR analysis

FT-IR spectroscopy was conducted using a Bruker Vertex 70 spectrometer (Billerica, USA) employing the KBr pellet method. Blank patches, riluzole-containing patches, and riluzole-PGD-containing patches of equal surface area were first solubilized completely in ethyl acetate. A volume of 50 μL of the resulting drug solution was then applied to the KBr pellets. After drying the samples at 50 °C for 3 min to evaporate the solvent, each sample was scanned 64 times in the wavenumber range of 400 to 4000 cm^−1^ to obtain the FTIR spectra (Li et al., 2017). The data were analyzed using Omnic software.

##### 2.3.1.3 DSC Study

The glass transition temperature (*T*_g_) of the PSA was measured via thermal analysis using a DSC − 1 instrument (Mettler Toledo, Greifensee, Switzerland) (Xu et al., 2024). Samples weighing 3 mg were accurately placed in aluminum pans, and the temperature was ramped from −80 to 25 °C at a heating rate of 2 °C/min. The *T*_*g*_ was determined from the DSC thermogram as the onset point of the inflection.

##### 2.3.1.4 Rheological study

The rheological properties of pure and drug-loaded PSA were assessed using a AR 2000 ex rheometer (TA Instruments, New Castle, DE, USA). Samples were placed between parallel stainless steel plates with an 8 mm diameter. The tests were conducted at 32 °C, corresponding to the temperature of human skin, and were repeated thrice. Oscillatory strain sweep measurements were performed within a strain range of 0.1–100 % at a frequency of 6.28 rad/s (1.0 Hz). The linear viscoelastic region (LVR) was identified as the strain range where the storage modulus (*G*') remained within 90 % of its initial value. Frequency sweep tests were then conducted across a frequency range of 0.1–100 rad/s, maintaining a controlled strain of 10 %, based on the LVR determined from the strain sweep. Creep tests were performed by applying a constant stress of 200 Pa to the samples for 180 s, followed by a recovery period of 540 s. During these tests, the strain values were kept within 90 % of the LVR, and creep and recovery curves were recorded (Liu et al., 2020).

##### 2.3.1.5 Cold flow study

The cold flow characteristics were evaluated using 10 mm diameter circular specimens punched from PSA patches. After removing the release liner, the PSA-coated surface was carefully placed at the center of a clean microscope slide to ensure wrinkle-free application. A fluoropolymer-coated backing film (Scotchpak **™** 9720) was then applied with the coated side facing downward to fully cover the slide surface.

Cold flow was induced by applying a 1 kg stainless steel weight onto the test assembly. The samples were maintained at 32 °C for 24 h to simulate skin surface temperature. The cold flow percentage was calculated according to Eq. (4) as the weight ratio between flowed and unflowed PSA specimens (Krishnaiah et al., 2014; Li et al., 2024).

(4) $$\text{Cold flow}\left(\%\right)=\left[\left(W_{C}-W_{0}\right)/W_{0}\right]\times 100\%$$

where *W*c represents the weight of cold flow-treated samples and *W*_0_ denotes the initial weight of untreated samples.

##### 2.3.1.6 ^13^C NMR spectra of patch

The molecular interactions and binding sites among riluzole, PGD, and semi-IPN PSA were in*v*estigated using ^13^C NMR spectroscopy. An A*v*ance III 600 MHz NMR spectrometer (Bruker, Germany)(Zhao et al., 2017) was utilized to assess chemical shifts (ppm) referenced to tetramethylsilane (TMS) in CDCl_3_. For preparing the samples, each sample was also solubilized in deuterated chloroform (CDCl_3_).

##### 2.3.1.7 Molecular docking

The molecular structures of semi-IPN PSA, riluzole, and PGD were drawn using ChemDraw *v*ersion 22.0.0. Molecular docking simulations were conducted with Materials Studio software (version 7.0, Accelrys, San Diego, USA). Initial geometry optimization of each component was performed using the Forcite module, followed by docking studies in the Blend module. Structural refinement and interaction analysis were conducted using the COMPASS II force field to evaluate potential binding conformations and interaction energies between the drug and polymer matrices (Sun et al., 2023). The optimized structure of semi-IPN PSA was individually paired with riluzole and PGD within a unified molecular model to assess potential intermolecular interactions. Hydrogen bond distances between functional groups were measured to evaluate the strength and nature of these interactions.

#### 2.3.2 Percutaneous drug absorption process

##### 2.3.2.1 Percutaneous penetration in solution

The transdermal absorption study of the solution formulations was performed using a vertical diffusion cell. Three types of samples were prepared by dissolving the drugs in an ODO (oleyl alcohol) solution: (1) blank ODO solution, (2) ODO solution containing 10 % (*w*/*v*) riluzole, and (3) ODO solution containing 17 % (w/v) riluzole and 10 % (w/v) PGD. At the start of the experiment, each formulation was applied to the SC side of the excised skin. All other experimental conditions, including sampling intervals, were identical to those used in the in vitro permeation studies.

##### 2.3.2.2 Tape Stripping Transdermal Experiment

The SC was completely removed by repeatedly stripping the skin 20 times using 2.5 cm-wide polyvinyl chloride (PVC) tape. The resulting SC-depleted skin samples were mounted in a horizontal single-chamber diffusion cell for a 24-h transdermal permeation study. Transdermal patches, with or without PGD, were applied to the stripped skin, and the same procedure was also conducted on unstripped skin with intact SC as a control. All other experimental parameters, including sampling intervals and analytical methods, were consistent with those used in the standard in vitro transdermal permeation experiments. This study was designed to evaluate the role of the SC as the primary barrier to drug penetration(Zhang et al., 2021b).

##### 2.3.2.3 In vitro quantification of drug retention within full-thickness skin following transdermal application

To evaluate the in vitro retention of riluzole within the entire skin, samples were collected after 24 h of transdermal permeation(Zhang et al., 2021b). Upon completion of the experiment, the skin was carefully removed from the diffusion cell, and any surface residue of solvent or drug was gently wiped away using cotton. The cleaned skin was then cut into small fragments and transferred into EP tubes. Methanol was added to extract the retained drug, followed by sonication for approximately 1 h. After sonication, the skin fragments were discarded, and the remaining solution was centrifuged at 16,000 rpm for 5 min to eliminate insoluble matter. The supernatant was diluted with methanol as needed and subsequently analyzed using HPLC (Sarkar et al., 2018).

##### 2.3.2.4 ^13^C NMR analysis

The interaction strength among ceramide NS (common lipid in skin), riluzole, and PGD was evaluated using ^13^C NMR spectroscopy. Accurately weighed amounts of each compound were individually solubilized in CDCl_3_, and the resulting solutions were transferred to NMR tubes. TMS was used as an external reference standard. Spectral data were acquired using a Bruker 600 MHz NMR spectrometer, and the corresponding ^13^C chemical shifts were recorded to assess potential molecular interactions.

##### 2.3.2.5 CLSM Study

CLSM was employed to evaluate the effect of PGD on enhancing transdermal drug penetration. A supersaturated solution of Nile red was utilized as a model fluorescent probe in place of riluzole. The skin pretreatment protocols and experimental conditions for both the PGD-treated and control groups were identical to those described in Section 2.3.2.1. Following a 1-h application, the skin was thoroughly rinsed with distilled water and gently dried using filter paper. The treated skin samples were then mounted between a glass slide and a coverslip for imaging.

CLSM imaging was conducted using an LSM 710 laser scanning confocal microscope (Carl Zeiss AG, Jena, Germany). Nile red was excited at a wavelength of 514 nm, and fluorescence emission was detected within the 539–793 nm range. *Z*-stack images were acquired across the full thickness of the skin, from the SC to the dermis (Cui et al., 2015). Subsequent image processing was carried out using ZEISS ZEN 2012 software.

##### 2.3.2.6 ATR-FTIR analysis

ATR-FTIR spectroscopy was employed to examine not only the influence of drugs and CPEs on the skin but also to assess changes in the structural organization of skin lipids and keratin following their application. Four sample groups were prepared: (1) blank ODO solution, (2) ODO solution containing riluzole, and (3) ODO solution containing both riluzole and PGD. After a 2-h treatment of rat skin with each solution, the skin was carefully remo*v*ed from the diffusion cells and gently cleaned with cotton to eliminate residual formulation. The samples were then positioned with the SC side in direct contact with a ZnSe crystal for analysis. Infrared spectra were recorded using a NEXUS +70 spectrometer (Thermo Electron Corporation, Waltham, MA, USA) over a wavenumber range of 4000–500 cm^−1^.

##### 2.3.2.7 Raman analysis

Raman spectroscopy was utilized to investigate the interaction mechanisms between the drug, PGD and skin components. Spectral measurements were conducted using a Renishaw inVia Laser Micro-Raman spectrometer equipped with a 633 nm laser source operating at a power of 300 mW. Spectra were acquired over the wavenumber range of 4000–300 cm^−1^ with an acquisition time of 1 s. Subsequent data analysis and peak deconvolution were carried out using PeakFit 4.0 software. (San Jose, USA).

##### 2.3.2.8 SAXS Experiment

Small-angle X-ray scattering (SAXS) was employed to investigate the molecular interactions among the drug, PGD, and SC lipids. Rat abdominal skin was incubated in a physiological saline solution (pH 4.0) containing 10 % (*w*/*v*) trypsin at 37 °C for 24 h to facilitate enzymatic separation of skin layers. The SC was carefully isolated using tweezers, rinsed thoroughly with purified water to remove residual enzymes, gently blotted dry with cotton, and subsequently vacuum-dried for analysis (Tian et al., 2021). Drug solutions were prepared using ethanol as the solvent, including formulations of riluzole alone and riluzole combined with PGD. The isolated SC samples were treated with 1 mL of each drug solution. After drying, the treated skin specimens were subjected to SAXS analysis using an Anton Paar SAXS instrument (Graz, Austria) under the following conditions: 25 °C, 40 kV/50 mA, and an exposure time of 900 s. The lamellar repeat distance of SC lipid bilayers was calculated based on the diffraction data using Bragg's law:

(5) $$S=\left(\frac{2}{\lambda }\right)∙\sin\theta$$

(6) 2dsinθ=nλ

Where d (d = 5.838 nm) is the lamellar repeat distance of silver behenate, 2θ is the scattering angle (Horita et al., 2015), S is the lattice spacing, and n represents the diffraction order.

##### 2.3.2.9 Molecular modeling

Molecular docking was employed to further elucidate the interaction strengths among riluzole, the riluzole–PGD complex, and ceramide NS. As the most abundant lipid component in the SC, ceramide NS was selected as a representative model for evaluating interactions with skin lipids. Drug molecular structures were sourced from the ChemicalBook database and redrawn using ChemDraw 22.0.0. Docking simulations were carried out using Materials Studio software (version 7.0, Corrys, San Diego, USA). Structural optimization of each component was initially performed using the Forcite module, followed by molecular docking in the Blend module. Final geometry optimization of the resulting complexes was conducted using the COMPASS II force field (Adeleke et al., 2016). Molecular dynamics simulations were employed to construct cubic simulation systems comprising the drug, CPE, and ceramide using the Amorphous Cell module. The cohesive energy density (CED) of each system was subsequently calculated.

### 2.4 Statistical analysis

The data were statistically analyzed using ANOVA, with the results expressed as mean ± standard deviation. The significance level was set at *p* < 0.05.

## 3 Results

### 3.1 Optimization of prescription

#### 3.1.1 Influence of riluzole loading on skin penetration

**Fig. 2:**
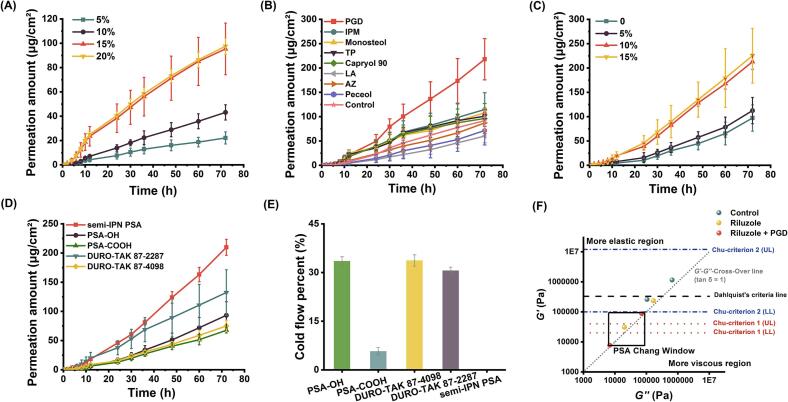
(A) Skin penetration curve of patches with different drug loadings (*n* = 4), (B) In vitro skin penetration of patches with different CPEs (*n* = 4). (C) Skin penetration curve of patches with different CPE amounts (*n* = 4). (D) Skin penetration curve of patches with the different PSAs (*n* = 4). (E) Cold flow percent of different PSAs. (F) The modulus of pure semi-IPN PSA, riluzole-semi-IPN PSA, riluzole-PGD-semi-IPN PSA (Mean ± SD, *n* = 3); In parentheses: *ω*, unit rad/s; UL indicates the upper limit. LL Indicates the lower limit.

The transdermal permeation behavior of riluzole was evaluated at varying drug loadings, as illustrated in Fig. 2**A**. A gradual increase in cumulative permeation was observed with increasing drug concentrations from 5 % to 20 % (*w*/w). Importantly, the formulation containing 20 % riluzole achieved a cumulative permeation of 97.54 ± 6.81 μg/cm^2^, which was not significantly different (*p* > 0.05) from that of the 15 % formulation (95.35 ± 2.22 μg/cm^2^). Based on these findings, the 15 % drug loading was selected for subsequent investigations.

#### 3.1.2 Influence of CPE on skin penetration

As depicted in Fig. 2B, PGD exhibited the strongest transdermal permeation-enhancing effect, with a cumulative permeation of 218 ± 7.83 μg/cm^2^, followed by IPM. The enhancement effects of the remaining CPEs were not statistically significant. Therefore, PGD was selected as the most effective enhancer for further formulation development. Fig. 2C illustrates the impact of PGD concentration on drug permeation, revealing the following order: 15 % > 10 % > 5 % > 0 %. However, no statistically significant difference was observed between the 10 % and 15 % concentrations. Given the potential risk of skin irritation associated with higher enhancer concentrations, 10 % PGD was selected as the optimal level for continued formulation screening.

#### 3.1.3 Influence of PSAs on skin penetration

Five acrylic-based pressure-sensitive adhesives (PSAs) were evaluated to assess the impact of various functional groups on transdermal drug delivery. As shown in Fig. 2D, the transdermal permeation followed the order: semi-IPN PSA > 87–2287 > PSA-OH > 87–4098 > PSA-COOH. Among these, the semi-IPN PSA exhibited the highest permeation efficiency. Therefore, it was selected for further formulation development.

#### 3.1.4 Cold flow study

As shown in Fig. 2E, the cold flow percentages for PSA-OH, PSA-COOH, 87–4098, and 87–2287 were 33.55 %, 5.76 %, 33.73 %, and 30.62 %, respectively, whereas the semi-IPN PSA exhibited no detectable cold flow. Despite its low cold flow, PSA-COOH demonstrated poor adhesive performance and a relatively rigid texture. These findings indicate that the semi-interpenetrating network structure of the semi-IPN PSA substantially enhances its cohesive strength, making it particularly well-suited for transdermal patch formulations with high drug-loading capacity.

#### 3.1.5 Rheological study

**Fig. 3:**
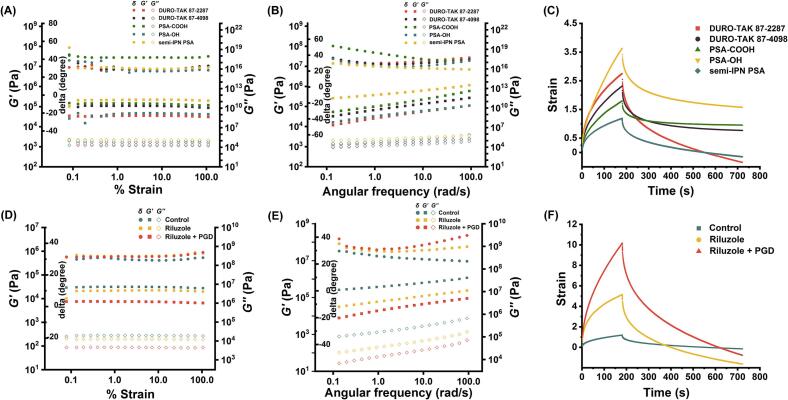
(A) Strain sweep of different PSAs. (B) Frequency sweep of different PSAs. (C) Creep sweep of different PSAs. (D) Strain sweep between semi-IPN PSA, riluzole-semi-IPN PSA, and riluzole-PGD-semi-IPN PSA. (E) Frequency sweep between semi-IPN PSA, riluzole-semi-IPN PSA, and riluzole-PGD-semi-IPN PSA. (F) Creep sweep between semi-IPN PSA, riluzole-semi-IPN PSA and riluzole-PGD-semi-IPN PSA.

Due to their viscoelastic properties, PSAs can exhibit both solid-like and liquid-like behaviors, and their flow characteristics can be evaluated by measuring viscoelastic parameters (Xu et al., 2024). In the strain sweep test, rheological data (Fig. 3A) revealed that the pure semi-IPN PSA exhibited dominant elastic behavior (*G′ > G″*), indicating a more pronounced solid-like character compared to conventional PSAs. This structural advantage translates into improved cohesive strength and resistance to cold flow, making the semi-IPN PSA particularly suitable for TDDSs with high drug loading. In the frequency sweep test, the semi-IPN PSA demonstrated significantly improved viscoelastic properties, with a higher storage modulus (*G′*) and lower loss tangent (δ) values across the frequency range of 0.1–100 rad/s (Fig. 3B), confirming the formation of a robust semi-interpenetrating network. This reinforced structure enhances mechanical stability and minimizes the plasticizing effects induced by drug and CPEs—an essential feature for maintaining performance in high-load transdermal formulations. At 0.1 rad/s, the *G′* values for the semi-IPN PSA, PSA-COOH, 87–4098, PSA-OH, and 87–2287 were 259,300, 53,550, 32,640, 16,250, and 11,730 Pa, respectively. Furthermore, creep analysis (Fig. 3C) demonstrated the semi-IPN PSA's improved mechanical integrity, as evidenced by significantly lower compliance compared to conventional PSAs (*p < 0.01*). This resistance to deformation under sustained stress further underscores the increased cohesive strength of the semi-IPN system, validating its suitability for high-performance transdermal patch applications.

When riluzole and PGD were incorporated into the semi-IPN PSA, the adhesive maintained its structural integrity across the 0.1–100 rad/s range in the strain sweep test (Fig. 3D), indicating favorable viscoelastic properties for transdermal drug delivery applications. The absence of deformation under strain suggests that the formulation retains sufficient mechanical strength to ensure functional stability during use.

In the frequency sweep analysis, the addition of riluzole and PGD resulted in pronounced plasticization effects on the PSA matrix. At 0.1 rad/s (Fig. 3E), compared to the unmodified PSA (*δ* = 29.61°, *G′* = 259,300 Pa), the incorporation of riluzole led to an increase in *δ* to 35.13° and a decrease in *G′* to 31,060 Pa. This plasticizing effect was further intensified by the combination of riluzole and PGD (*δ* = 38.74°, *G′* = 7761 Pa). These changes reflect increased molecular mobility and reduced intermolecular cohesion, likely due to the occupation of polymer free-volume by small molecules, which disrupts the internal network structure. This alteration in viscoelastic behavior supports increased drug diffusion, consistent with a classic plasticization mechanism that facilitates transdermal drug release. Creep testing further confirmed the plasticizing influence of PGD, as its inclusion significantly increased the creep compliance of the semi-IPN PSA (Fig. 3F), indicating higher material deformability. This increase in fluidity is attributed to PGD's ability to weaken intermolecular forces and promote polymer chain mobility, improving drug release efficiency from the matrix.

As shown in Fig. 2F, all semi-IPN PSA-based formulations satisfied Dahlquist's criterion, indicating adequate tack performance suitable for skin adhesion (Luo et al., 2020). Frequency sweep analysis over the 0.1–100 rad/s range further confirmed elastic-dominant behavior in all groups, as evidenced by tan *δ* values consistently below 1. The inclusion of both riluzole and PGD resulted in a decrease in the storage modulus (*G′*) of the semi-IPN PSA (*p <* 0.01). Despite this reduction, the semi-IPN PSA maintained viscoelastic properties within Chang's universal PSA window, demonstrating an optimal balance between adhesive strength and drug release capability—essential for high-performance TDDS (Wolff and Irsan, 2014).

#### 3.1.6 Optimization of formulation

Through single-factor experiments, the optimal drug loading, type and amount of CPE, and type of PSA were determined for the patch formulation. Skin permeation amount was selected as the response variable (*Y*), to maximize this parameter. To further optimize the formulation, a three-factor, three-level BBD was employed, with riluzole content (*X*_1_), PGD content (*X*_2_), and patch thickness (*X*_3_) as the independent variables; The corresponding ranges were as follows; patch thickness (80–140 μm), drug loading (10 %–20 %), and CPE content (5 %–15 %). The optimization process was carried out using Design Expert® software, and the resulting parameters are presented in Table 1. The BBD, incorporating riluzole content (*X*_1_), PGD content (*X*_2_), and patch thickness (*X*_3_), along with the experimental results for the skin permeation amount (*Y*), are presented in Table 2. Finally, the software simulation yielded an equation for the coded factors.

**Table 1:** Factor codes and levels for BBDs.

Level	Riluzole content (*X*_1_, %)	PGD content (*X*_2_, %)	Patch thickness (*X*_3_, μm)
−1	10	5	80
0	15	10	110
1	20	15	140

**Table 2:** Coding BBD (riluzole content *X*_1_, PGD content *X*_2,_ and patch thickness *X*_3_) and experimental results (the skin permeation amount *Y*).

Run	*X*_1_	*X*_2_	*X*_3_	*Y*
1	−1	0	−1	98.23
2	−1	−1	0	94.21
3	−1	1	0	96.24
4	−1	0	1	102.91
5	0	0	0	225.26
6	0	0	0	216.57
7	0	0	0	214.65
8	0	−1	1	120.56
9	0	0	0	219.56
10	0	1	−1	125.72
11	0	1	1	120.17
12	0	0	0	222.95
13	0	-1	-1	102.73
14	1	-1	0	130.99
15	1	1	0	156.23
16	1	0	1	167.67
17	1	0	-1	163.24

*Y* = 219.80 + 28.32 × _1_ + 6.23 × _2_ + 2.67 × _3_ + 5.80 *X*_1_*X*_2_–0.0625 *X*_1_*X*_3_–5.84 *X*_2_*X*_3_–42.33 × _1_^2^–58.05 × _2_^2^–44.45 × _3_^2^.

This equation was utilized to model the relationship between the three variables and the transdermal permeation response. The equation structure was analyzed as follows: *Y* represents the response variable (skin permeation amount, μg/cm^2^). *X*_1_, *X*_2_, and *X*_3_ are coded independent variables. The constant term (219.80) indicates the predicted permeation at the center point (*X*_1_ = 0, *X*_2_ = 0, *X*_3_ = 0). The first-order terms (28.32 × _1_, 6.23 × _2_, 2.67 × _3_) reflect main effects, showing linear influences of individual factors on *Y*. The interaction terms (5.80 *X*_1_*X*_2_, −0.0625 *X*_1_*X*_3_, −5.84 *X*_2_*X*_3_) represent synergistic effects between two factors. The quadratic terms (−42.33 × _1_^2^, −58.05 × _2_^2^, −44.45 × _3_^2^) demonstrate nonlinear effects, indicating inflection points or saturation trends. The optimization strategy focused on utilizing the *X*_1_*X*_2_ synergistic effect (+5.80) while avoiding thickness increases at high PGD levels (*X*_2_*X*_3_ = −5.84), with the *X*_1_*X*_3_ interaction effect (−0.0625) being negligible. All quadratic terms were negative (*X*_1_^2^/*X*_2_^2^/*X*_3_^2^), indicating bell-shaped response surfaces with distinct optima.

**Fig. 4:**
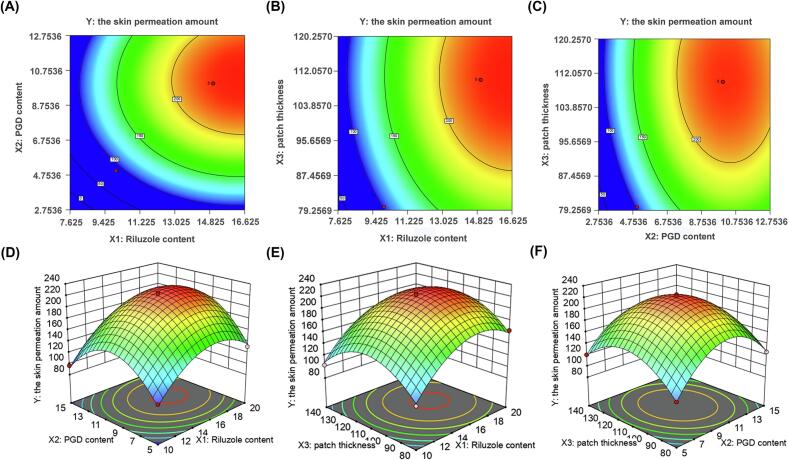
(A-C) Isoline map and (D—F) 3D surface map of the effects of riluzole content *X*_1_, PGD content *X*_2,_ and patch thickness *X*_3_ on the skin permeation amount *Y*.

The effects of each factor and their interactions are visually represented in the contour and 3D response surface plots (Fig. 4). The predictive model exhibited high statistical significance (*p* < 0.0001), confirming its reliability. Based on response surface analysis, the optimal formulation was predicted to contain 16.69 % loaded drug, 10.34 % PGD content, and a patch thickness of 110.75 μm. For practical implementation, the final formulation was adjusted to 17 % drug loading, 10 % PGD content, and 111 μm thickness. In vitro skin permeation studies showed a permeation value of 212.94 ± 3.41 μg/cm^2^, which was not significantly different from the model's predicted value of 224.85 μg/cm^2^ *(p* = 0.116), validating the model's accuracy. Accordingly, this optimized formulation was selected for further characterization and evaluation. The statistical values of *p* values are shown in Table 3.

**Table 3:** ANOVA of formulation factors affecting permeation.

Source of Variance	Sum of Squares	Mean Square	*F*-value	*p*-value
Model	40,681.54	3390.13	176.69	0.000
*X*_1_-Riluzole content	6415.05	6415.05	334.35	0.000
*X*_2_-PGD content	310.88	310.88	16.20	0.016
*X*_3_-Patch thickness	57.19	57.19	2.98	0.159
*X*_1_*X_2_	134.68	134.68	7.02	0.057
*X*_1_*X_3_	0.016	0.016	0.001	0.979
*X*_2_*X_3_	136.66	136.66	7.12	0.056
Error	76.75	19.19		
Total	40,758.29			

#### 3.1.7 Pharmacokinetics study

**Fig. 5:**
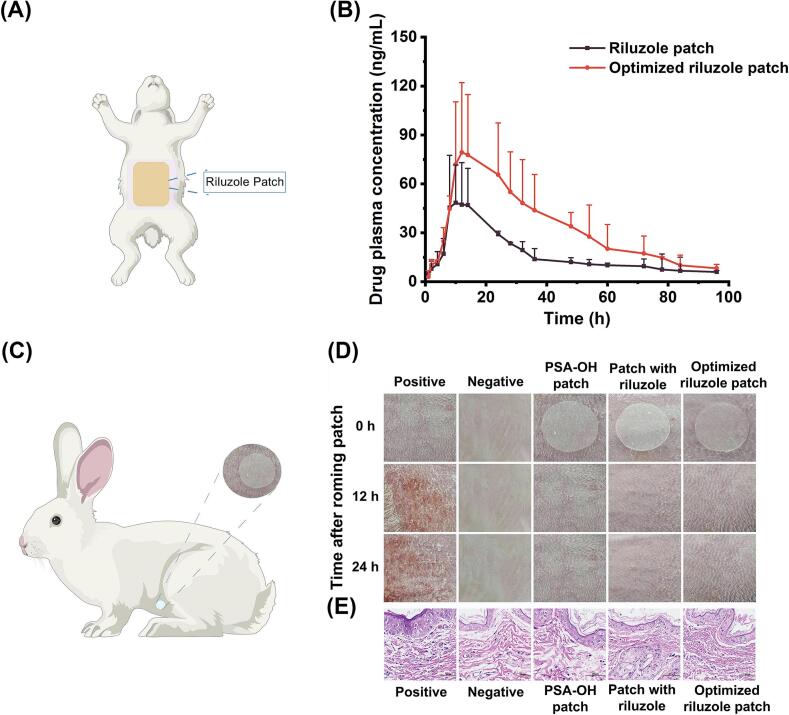
(A) Pharmacokinetic experiment of rabbit skin drug administration diagram. (B) Plasma concentration curve of patch groups (riluzole patch group and optimized patch group). (C) Schematic diagram of rabbit skin administration for skin stimulation test. (D) Skin irritation test and H&E staining of the positive group, negative group, blank patch group, riluzole patch group and optimized riluzole patch group.

Pharmacokinetics provides valuable insights into the dynamic behavior of drugs within biological systems. In this study, pharmacokinetic analysis was employed to assess the in vivo transdermal delivery performance of the optimized patch formulation. The plasma concentration-time profiles of both the riluzole-containing patch and the optimized riluzole-PGD-containing patch are presented in Fig. 5B, with corresponding pharmacokinetic parameters summarized in Table 4. At an equivalent dosage, the optimized patch achieved a more rapid and pronounced increase in the maximum plasma concentration (*C*_max_) of riluzole. The area under the concentration-time curve (AUC_0–t_) for the optimized formulation reached 3548.61 ± 1597.56 h·ng/mL, substantially exceeding that of the unoptimized riluzole patch. This improvement is attributed to the incorporation of PGD, which facilitated increased systemic absorption, resulting in more stable plasma levels and a prolonged mean residence time (MRT_0–t_). The optimized patch exhibited extended half-life and MRT values compared to the control, indicating sustained drug release and prolonged therapeutic action. Compared with orally administered formulation, these pharmacokinetic advantages support the potential of the optimized patch to reduce dosing frequency and improve patient compliance through improved delivery efficiency. The relative bioavailability and absolute bioavailability of the optimized riluzole patch were obtained as 185.79 % and 1.56 %, respectively. The absolute bioavailability of patch is relatively low. For instance, the absolute bioavailability of the fluorescein isothiocyanate-dextran patch is 0.54 % (Hoyer et al., 2009). the absolute bioavailability of tolterodin patch is 6.3 % (Liu et al., 2011). Riluzole has 2 hydrogen bond donors and 3 hydrogen bond acceptors, and has strong interaction with the skin and PSA. After adding the CPE, the relative bioavailability has been improved.

**Table 4:** Specific pharmacokinetic parameters of the optimized riluzole patch group with PGD, the riluzole patch group without PGD, the intravenous injection group, and oral gavage group.

Pharmacokinetic Parameters	Optimized riluzole patch	Riluzole patch	Riluzole solution (i.v.)	Riluzole solution (i.g.)
AUC_0-∞_ (ng/mL*h)	3548.61 ± 1597.56	1910.04 ± 523.12	7157.03 ± 698.99	3334.03 ± 1002.45
MRT_0-t_ (h)	34.91 ± 11.31	33.61 ± 8.62	0.57 ± 0.03	17.01 ± 6.22
*t*_1/2_ (h)	49.36 ± 5.26	40.68 ± 1.25	7.95 ± 1.23	18.28 ± 3.46
*T*_max_ (h)	12.02 ± 0.89	10.13 ± 1.47	–	6.05 ± 0.52
*C*_max_ (ng/mL)	74.34 ± 13.62	48.41 ± 12.38	–	199.83 ± 25.11

#### 3.1.8 Skin irritation tests and H&E staining

The skin irritation study, performed using the abdominal skin of rabbits aimed to evaluate the potential irritant effects associated with the formulation components, confirming the patch's suitability for safe and non-allergenic application in transdermal delivery (Jiang et al., 2025). Fig. 5D presents the skin appearance at various time points following patch application. The optimized patch group exhibited excellent dermal compatibility throughout the observation period, with no signs of irritation such as erythema or edema. However, the positive control group showed significant skin reactions, including pronounced erythema and edema that progressed to scab formation, indicating severe irritation. Histological analysis via H&E staining (Fig. 5E) further supported these observations. While the positive control group displayed significant epidermal hyperpigmentation, no inflammatory cell infiltration or structural abnormalities were observed in the skin tissues of any patch-treated groups. Similarly, no significant histopathological differences were found compared to the negative control. The results demonstrate that the optimized patch is well-tolerated and safe for transdermal application.

### 3.2 Mechanism of the Enhancement effect of PGD

#### 3.2.1 Drug release process

##### 3.2.1.1 In vitro drug release of patch

**Fig. 6:**
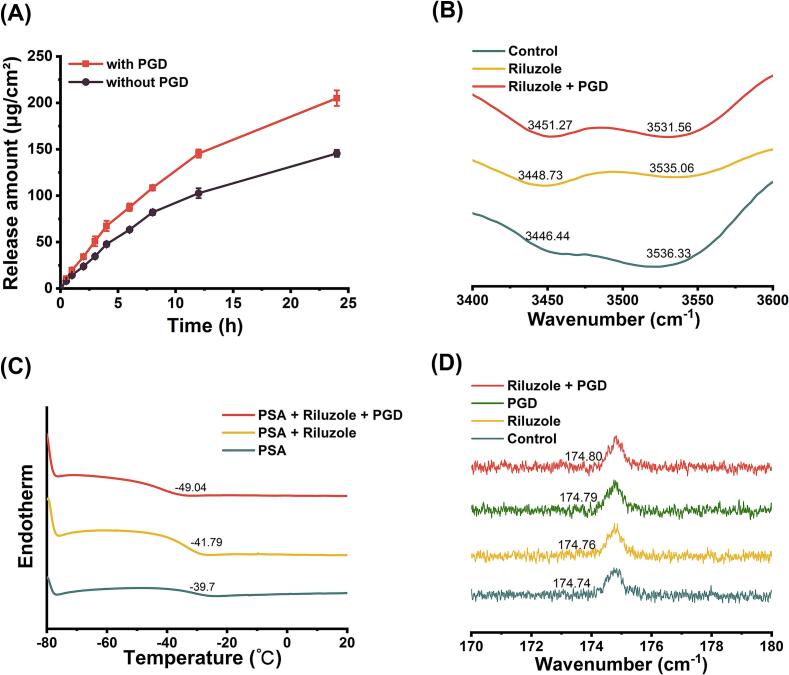
(A) In vitro release of patches with or without PGD (*n* = 4). (B) FT-IR (C) DSC spectra of semi-IPN PSA, riluzole-semi-IPN PSA, riluzole-PGD-semi-IPN PSA. (D) ^13^C NMR spectra of semi-IPN PSA, riluzole-semi-IPN PSA, PGD-semi-IPN PSA, riluzole-PGD-semi-IPN PSA.

In vitro release studies were performed to assess the influence of PGD on the release behavior of riluzole from transdermal patches. As shown in Fig. 6A, the inclusion of 10 % PGD evidently increased the cumulative release of riluzole over 24 h. The patches containing PGD achieved a cumulative release of 205.03 ± 8.40 μg/cm^2^, compared to 145.55 ± 3.70 μg/cm^2^ for the patches without PGD. These findings highlight the critical role of PGD in facilitating drug diffusion and effectively modulating the release kinetics of riluzole from the patch matrix.

##### 3.2.1.2 FT-IR analysis

FT-IR spectroscopy was employed to investigate the molecular interactions among riluzole, PGD, and the semi-IPN PSA matrix. As depicted in Fig. 6B, the blank semi-IPN PSA displayed characteristic absorption bands at 3446.44 cm^−1^ and 3536.33 cm^−1^, respectively corresponding to C=O overtone and O—H stretching vibrations. Upon incorporation of riluzole (3448.73 cm^−1^, 3535.06 cm^−1^) and the riluzole–PGD mixture (3451.27 cm^−1^, 3531.56 cm^−1^), these peaks shifted significantly, indicating the existence of hydrogen bonds and dipole-dipole interactions among the components. The more substantial shifts observed in the riluzole–PGD mixture suggest that PGD forms stronger hydrogen bonds with the semi-IPN PSA than riluzole alone, highlighting its improved interaction potential within the formulation.

##### 3.2.1.3 DSC analysis

The *T*_*g*_, determined via thermal analysis, serves as a key indicator of polymer chain mobility and provides insight into the influence of CPEs on the thermodynamic behavior of PSAs. A lower *T*_*g*_ typically reflects greater molecular mobility, which facilitates drug diffusion through the polymer matrix. As shown in Fig. 6C, the *T*_*g*_ of the blank semi-IPN PSA was recorded at −39.7 °C. The incorporation of riluzole alone reduced the *T*_*g*_ to −41.79 °C, while the addition of the riluzole–PGD mixture further lowered it to −49.04 °C. Consistent with previous study, the incorporation of Azone significantly reduced the *T*_g_ of PSAs from −43.19 °C to −52.92 °C (Song et al., 2016). This progressive decrease in *T*_*g*_ indicates that PGD significantly enhances polymer chain mobility, resulting in increased fluidity and improved drug diffusivity. These findings highlight the effectiveness of PGD as a CPE in the transdermal delivery system.

##### 3.2.1.4 ^13^C NMR spectroscopy of the patch

To gain deeper insight into the interaction sites and strengths among riluzole, PGD, and the semi-IPN PSA matrix, ^13^C NMR spectroscopy was performed. As depicted in Fig. 6D, the ester carbonyl carbon of the semi-IPN PSA displayed a characteristic resonance at 174.74 ppm. Upon incorporation of riluzole, PGD, and the riluzole–PGD combination, this peak exhibited progressive downfield shifts to 174.76 ppm, 174.79 ppm, and 174.80 ppm, respectively. These chemical shift changes, together with the analysis of hydroxyl group interactions, point to the formation of hydrogen bonds between the ester functionalities and the incorporated components. The reduced electron density around the ester carbon, evidenced by the downfield shifts, confirms significant molecular interactions between riluzole, PGD, and the PSA matrix.

##### 3.2.1.5 Molecular docking

**Fig. 7:**
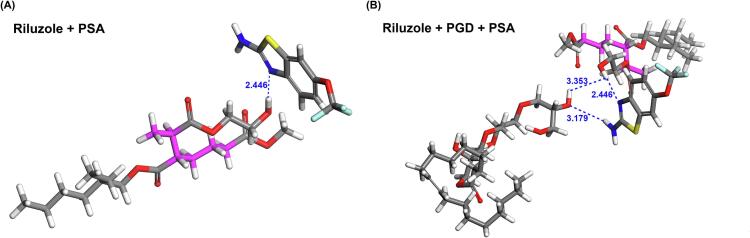
Molecular docking snapshot of (A) riluzole-PSA and (B) riluzole-PGD-PSA with minimal energy.

Molecular docking analysis was conducted to further corroborate the intermolecular interactions suggested by FTIR spectroscopy. The lowest energy conformations of the riluzole–PSA and riluzole–PGD–PSA complexes are presented in Fig. 7**.** As illustrated by the blue dashed lines, the distance of interaction between riluzole and PSA was measured at 2.446 Å, significantly shorter than the 3.353 Å distance observed between PGD and PSA. Hydrogen bonding typically occurs when the interaction distance is below 3.5 Å. These findings indicate the presence of hydrogen bonds in both cases, with a stronger interaction between riluzole and PSA. Interestingly, both PGD and riluzole appear to interact at the same binding site on the PSA, suggesting a competitive relationship. The presence of PGD disrupts the hydrogen bonding between riluzole and PSA, therefore weakening the interaction between them. These results support the hypothesis that PGD competes with riluzole for binding to PSA, potentially altering the drug-polymer interaction dynamics within the system.

#### 3.2.2 Percutaneous drug absorption

##### 3.2.2.1 Percutaneous penetration in solution

**Fig. 8:**
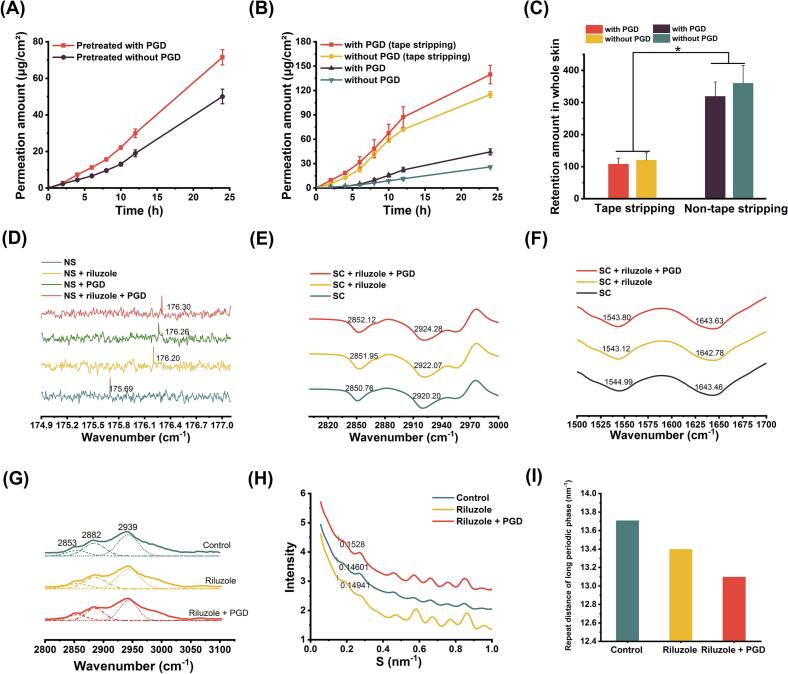
(A) Effect of PGD on 24 h transdermal absorption of riluzole in solution. (B) Penetration curve of 17 % riluzole patch with or without 10 % PGD. (C) In vitro retention of 17 % riluzole patch with or without 10 % PGD through peeled or untreated skin (*n* = 4). (D) ^13^C NMR of ceramide NS, ceramide NS-riluzole, ceramide NS-PGD and ceramide NS-riluzole-PGD. (*E*-F) ATR-FTIR spectrum of SC, SC-riluzole and SC-riluzole-PGD. (G) Raman spectrum of control skin, riluzole-skin and riluzole-PGD-skin. (H) SAXS spectrum of rat abdominal skin treated with control, riluzole, and riluzole-PGD. (I) LPP of control skin, riluzole-skin and riluzole-PGD-skin.

The transdermal permeation profiles of the drug solution systems are presented in Fig. 8A. The formulation lacking PGD achieved a cumulative permeation of 50.05 ± 4.00 μg/cm^2^, whereas the PGD-containing group reached a significantly higher value of 71.50 ± 4.14 μg/cm^2^. These results demonstrate that the inclusion of PGD substantially enhances the transdermal delivery of riluzole, highlighting its efficacy as a CPE in TDDS.

##### 3.2.2.2 Tape Stripping Transdermal Experiment

The barrier function of the SC was assessed using the tape-stripping method. To investigate the impact of this barrier on drug delivery, transdermal permeation studies were performed across four different groups of riluzole patch formulations.: (1) riluzole patch containing PGD applied to tape-stripped skin (with PGD (tape stripping, Q_1_)); (2) riluzole patch without PGD applied to tape-stripped skin (without PGD (tape stripping, Q_2_)); (3) riluzole patch containing PGD applied to intact skin (with PGD, Q_3_); and (4) riluzole patch without PGD applied to intact skin (without PGD, Q_4_).

As shown in Fig. 8B, the cumulative transdermal amount of riluzole over 24 h for groups Q_1_, Q_2_, Q_3_, and Q_4_ was 139.61 ± 11.45 μg/cm^2^, 115.13 ± 3.75 μg/cm^2^, 44.47 ± 3.78 μg/cm^2^, and 25.99 ± 1.28 μg/cm^2^, respectively. These results reveal several important insights: (1) Q_1_ > Q_3_ confirms that the SC is the principal barrier to transdermal drug absorption; (2) Q_4_ > Q_3_ suggests that PGD facilitates barrier disruption of the SC; and (3) Q_2_ > > Q_4_ indicates that PGD also promotes drug release from the patch matrix. These findings highlight the central role of the SC in limiting transdermal delivery and demonstrate the effectiveness of PGD as a CPE.

##### 3.2.2.3 In vitro skin retention of the drug across full-thickness skin

To further evaluate the barrier properties of the SC, an in vitro skin retention study was conducted using four experimental groups: Q_1_ (PGD, tape-stripped skin), Q_2_ (no PGD, tape-stripped skin), Q_3_ (PGD, intact skin), and Q_4_ (no PGD, intact skin). As illustrated in Fig. 8C, the drug retention results showed that Q_2_ > Q_1_ and Q_4_ > Q_3_, confirm the role of PGD in improving drug permeation. A quantitative assessment of riluzole retention across different skin layers was performed to determine its distribution and to further clarify the SC's contribution to transdermal delivery. Comparative transdermal permeation experiments with and without the SC demonstrated that groups Q_3_ and Q_4_ exhibited markedly higher cumulative permeation than Q_1_ and Q_2_, indicating that the SC significantly limits riluzole penetration. These results confirm that the SC functions as the primary barrier to transdermal absorption. Moreover, the data emphasize the effectiveness of PGD as a CPE in TDDSs.

##### 3.2.2.4 ^13^C NMR analysis

The molecular interactions between riluzole, PGD, and ceramide NS was further investigated using ^13^C NMR spectroscopy. As shown in Fig. 8D, the characteristic peak corresponding to the amide carbon of ceramide NS appeared at 175.69 ppm. Following treatment with riluzole, PGD, and the riluzole–PGD combination, this peak shifted to 176.20 ppm, 176.26 ppm, and 176.30 ppm, respectively. These progressive upfield shifts indicate the formation of hydrogen bonds between the amino group of ceramide NS and either riluzole or PGD, resulting in a reduced shielding effect on the carbon atom. The observed shifts confirm the presence of intermolecular interactions among riluzole, PGD, and ceramide NS.

##### 3.2.2.5 ATR-FTIR analysis

ATR-FTIR spectroscopy was employed to examine alterations in the structural organization of SC lipids and to assess the impact of riluzole and PGD treatment on skin lipid arrangement. The spectral peaks were assigned to the stretching vibrations of SC lipids (ν_as_ CH_2_, ≈ 2920 cm^−1^; ν_s_ CH_2_, ≈ 2850 cm^−1^), as well as the amide II (≈ 1543 cm^−1^) and amide I (≈ 1638 cm^−1^) bands of SC keratin (Zhang et al., 2021b). Fig. 8E and F demonstrate that the addition of various components did not produce any significant changes in the amide I and II bands (*p* > 0.05), suggesting that neither riluzole nor PGD interacted with keratin in the SC. The asymmetric CH_2_ stretching vibration band (*V*_as_ CH_2_) of untreated skin appeared at 2920.20 cm^−1^. Following treatment with riluzole and the riluzole-PGD formulation, the CH₂ asymmetric stretching band shifted to higher wavenumbers (2922.07 cm^−1^ and 2924.28 cm^−1^, respectively). These spectral changes reflect disruptions in lipid packing and increased fluidity within the SC, indicating that both riluzole and PGD interact with skin lipids. This interaction likely facilitates increased drug permeation by altering the structural organization of the lipid matrix, supporting the proposed role of PGD as an effective CPE in TDDSs.

##### 3.2.2.6 Raman analysis

To gain deeper insight into the interaction mechanisms of riluzole and the riluzole-PGD formulation with the skin, Raman spectroscopy was utilized. Characteristic Raman bands for SC lipids include the symmetric (ν_s_CH₂ ≈ 2850 cm^−1^) and asymmetric (ν_as_CH₂ ≈ 2880 cm^−1^) stretching vibrations of methylene groups. The ratio of the area under the curve (AUC) of these peaks—AUC(ν_as_CH_2_)/AUC(ν_s_CH_2_)—serves as an indicator of lipid chain order within the SC, with higher values reflecting a more ordered lipid arrangement. As illustrated in Fig. 8G, untreated skin exhibited a ratio of 2.34. After application of riluzole and the riluzole-PGD formulation, this ratio decreased to 2.22 and 2.11, respectively. These findings suggest that both treatments disrupt the ordered lipid architecture of the SC, facilitating increased transdermal drug permeation.

##### 3.2.2.7 SAXS experiment

SAXS was utilized to investigate the nanoscale organization of intercellular lipids within the SC. These lipids are arranged in well-defined lamellar phases, each characterized by a distinct repeat distance, reflecting their structural periodicity (Jia et al., 2024). The observed diffraction peaks correspond to the periodicity of the lamellar structures formed by intercellular lipids in the SC. These peaks, which include two partially overlapping signals, are attributed to the long and short lamellar phases. The repeat distances of these lamellar arrangements were determined using Bragg's law (Zhang et al., 2023b). The long-period phase (LPP) and short-period phase (SPP) represent two distinct lamellar arrangements within the intercellular lipids of the SC, typically exhibiting repeat distances of approximately 13 nm and 6 nm, respectively, as reported in previous literature. As shown in Fig. 8H and I, treatment with ethanolic solutions of riluzole and riluzole containing 10 % PGD resulted in a reduction of the LPP repeat distance from 13.70 nm to 13.39 nm and 13.09 nm, respectively, while the SPP distance remained stable at 6.011 nm. When considered alongside ATR-FTIR findings, these observations suggest that PGD perturbs the LPP structure without affecting the SPP, thus altering the lipid lamellae organization. This structural disruption likely contributes to enhanced skin permeability, offering molecular-level validation of PGD's function as a CPE in TDDS.

##### 3.2.2.8 CLSM analysis

**Fig. 9:**
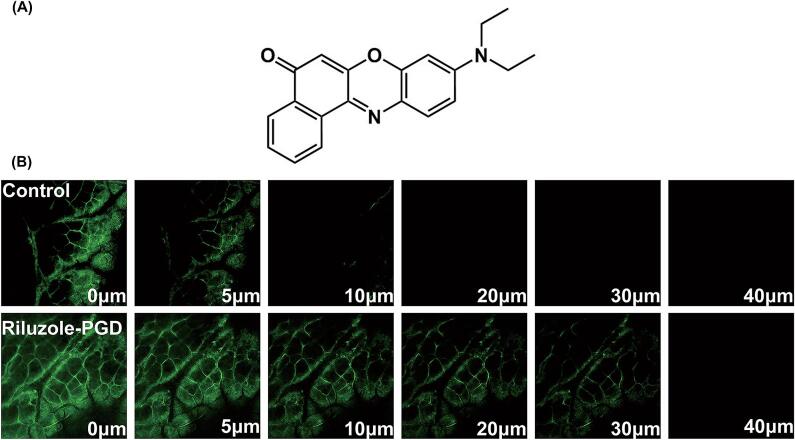
(A) The structure of Nile red. (B) Confocal laser scanning microscope image of different depths of riluzole and PGD-treated skin. (For interpretation of the references to colour in this figure legend, the reader is referred to the web version of this article.)

CLSM was utilized to investigate the drug's permeation profile and elucidate the underlying enhancement mechanism. Due to the non-fluorescent nature of riluzole, Nile red—a lipophilic fluorescent dye with comparable physicochemical characteristics—was employed as a model compound. Its chemical structure is shown in Fig. 9A. Representative x-z cross-sectional CLSM images for the control and riluzole-PGD–treated groups are displayed in Fig. 9B, with green fluorescence indicating the presence of Nile red. The surface of the SC exhibited the characteristic “brick-and-mortar” architecture, with the hexagonal corneocyte arrangement visible. The fluorescence distribution revealed that Nile red primarily penetrated via the intercellular lipid route. In the control group, the dye reached a depth of approximately 10 μm, whereas skin treated with riluzole-PGD showed a markedly enhanced penetration depth of 30 μm. These findings visually confirm that PGD facilitates deeper transdermal delivery, in line with the in vitro skin retention results, and substantiate its role as an effective enhancer of drug transport across the SC.

##### 3.2.2.9 Molecular modeling

**Fig. 10:**
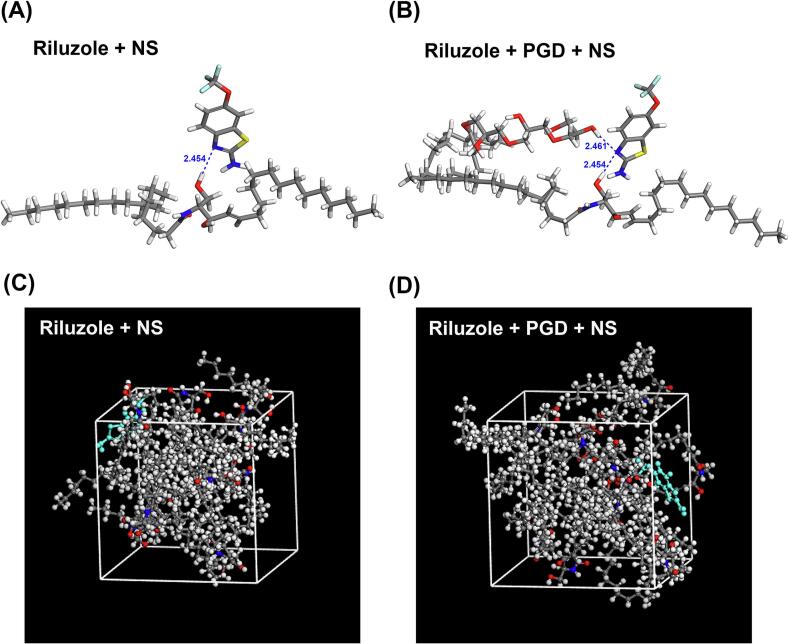
(A-B) Molecular docking snapshots and (C—D) post-simulation snapshots of riluzole-ceramide NS and riluzole-PGD-ceramide NS with minimum energy were obtained from molecular dynamics simulations.

To substantiate the intermolecular interaction results suggested by FTIR spectroscopy, molecular docking analyses were conducted. This approach enabled the evaluation of interaction strength between riluzole, PGD, and ceramide NS based on calculated intermolecular distances (Fig. 10). Molecular docking provided visual verification of the FTIR-predicted interactions, offering atomic-level resolution of the hydrogen bonding between riluzole's amino group and ceramide NS's hydroxyl group. The simulations overcame limitations of static characterization. The docking results revealed that the distance between riluzole and ceramide NS was 2.454 Å, which was slightly shorter than the 2.461 Å observed for the riluzole-PGD–ceramide NS complex, suggesting comparable but distinct interaction affinities.

Molecular dynamics simulations were performed to calculate the diffusion coefficient (*D*′) and the CED, providing further insight into the drug–lipid interactions. *D*′ is an indicator of drug mobility, where higher values correspond to enhanced diffusivity within the lipid matrix. CED quantifies the strength of interaction between the drug and ceramide NS, with elevated values reflecting stronger intermolecular interactions (Liu et al., 2017). The CED values for riluzole-NS and riluzole-PGD-NS were 3.24 × 10^8^ ± 1.62 × 10^6^ and 3.32 × 10^8^ ± 1.39 × 10^6^ kcal·mol^−1^, respectively, while the *D*′ values were 3.483 × 10^−2^ and 9.100 × 10^−**2**^ m^**2**^·s^−**1**^, respectively (Fig. 10). The results demonstrate that PGD enhances diffusion of riluzole molecules in skin by increasing the fluidity of skin lipids, which is consistent with the results of percutaneous penetration in solution. PGD might decrease the skin barrier.

## 4 Discussion

This study successfully developed a 72-h sustained-release riluzole transdermal patch that markedly improved transdermal delivery and pharmacokinetic performance through the combined action of a CPE and semi-IPN PSA. PGD exhibited significant skin barrier-modulating capabilities and effective permeation-enhancing activity, by selectively disrupting the lipid architecture of the SC with minimal interference with keratin (Wang et al., 2022). ATR-FTIR and Raman spectroscopy revealed shifts in the ν_s_CH_2_ and ν_as_CH_2_ stretching vibrations of SC lipids following PGD treatment, indicating a transition from a highly ordered to a more disordered alkyl chain arrangement. This disruption reduces diffusional resistance, consistent with previously reported lipid dynamics (Hathout et al., 2010). Hydrogen bonding interactions were observed between the PGD and skin lipids, as confirmed by ^13^C NMR analysis, which demonstrated that PGD forms hydrogen bonds with the amide carbonyl group of ceramide NS (Fig. 8D). This interaction likely disrupts the cohesive forces within the lipid matrix of the SC. This mechanism is comparable to the action of glycerol monooleate, which has been shown to reduce the LPP repeat distance from 13.70 nm to 13.09 nm via SAXS analysis. PGD did not alter the amide I/II bands of SC proteins, suggesting minimal impact on the secondary structure of keratin. This selective disruption of lipids without affecting protein conformation highlights PGD's lipid-specific mode of action and supports its low potential for skin irritation (Lane, 2013; Sun et al., 2023), reinforcing its suitability as a safe and effective surfactant-based permeation enhancer. The barrier properties of the SC are primarily upheld by tightly organized lipid bilayers, which are composed of ceramides, cholesterol, and free fatty acids (Uchida and Park, 2021). Structural studies have demonstrated that the extended acyl chains of ceramides interdigitate into the hydrophobic domains of the lipid matrix, stabilizing the bilayer architecture (Skolova et al., 2013). Cholesterol plays a crucial role in modulating the lamellar structures of the skin by increasing bilayer thickness and reducing membrane conductivity, both of which are essential for maintaining the integrity of the barrier function (Wennberg et al., 2012). As an amphiphilic molecule, PGD likely increases permeation through a dual mechanism: its octanoate moiety intercalates into the hydrophobic regions of the lipid matrix, while the propylene glycol moiety disrupts the organization of polar head groups through hydrogen bonding, thus increasing lipid fluidity. This mechanism is similar to, but more moderate than, the action of ethanol (Mistry and Notman, 2024). However, conventional CPEs, such as laurocapram, may affect both lipids and proteins simultaneously, which can lead to a higher potential for irritation.

PGD improved the release rate of riluzole (205.03 ± 8.40 μg/cm^2^, Fig. 6A), primarily through influnced intermolecular interactions. FT-IR analysis (Fig. 6B) showed redshifts in the C=O and O—H stretching vibrations of the semi-IPN PSA, suggesting that PGD functions as a “molecular bridge”. This interaction disrupts existing hydrogen bonds and dipole-dipole interactions among riluzole molecules and PSA polymer chains, reducing cohesive forces and promoting drug diffusion. Furthermore, ^13^C NMR analysis (Fig. 6D) revealed chemical shift changes in the ester carbonyl regions of PSA following PGD incorporation, indicating altered electronic environments. These findings support a mechanism in which PGD competes for riluzole-binding sites within hydrophobic acrylate microdomains, increasing the proportion of unbound drug available for diffusion and accelerating release kinetics (Seedher and Agarwal, 2013). Conventional PSAs, such as silicone-based adhesives, often hinder drug diffusion due to their high crystallinity. However, the dynamically cross-linked architecture of the semi-IPN PSA allows PGD to intercalate between polymer chains (Fig. 6C), as evidenced by the reduction in glass *T*_*g*_ observed in DSC analysis. This structural flexibility facilitates drug mobility while preserving sufficient viscoelasticity to meet the Dahlquist criterion for skin adhesion (Li et al., 2024).

Rheological modulation guided by free volume theory demonstrated that PGD functions as an effective plasticizer within the PSA matrix (Figs. 2F, 3A–F). DSC analysis showed a reduction in the glass *T*_*g*_, aligning with the plasticization behavior predicted by the Fox equation. The small molecular size of PGD allows it to occupy interstitial spaces between polymer chains, weakening interchain van der Waals interactions and enhancing segmental mobility (Fig. 6C).

(7) $$T_{g}=\frac{0.455∙z∙\langle D_{0}\rangle }{R}$$

To further investigate the factors affecting *T*_*g*_, a qualitative analysis was conducted (Luo et al., 2020), in which *T*_*g*_ is inversely related to free volume (via the coordination number, z) and directly proportional to the total interaction energy (*D*_0_) between polymer chain atoms. This model suggests that reductions in *T*_*g*_ correspond to increased free volume and decreased intermolecular interactions. Upon incorporation of PGD, the increased stochastic thermal motion of semi-IPN PSA chains led to a lower *T*_*g*_, promoting free volume expansion and enhancing molecular mobility within the polymer network. These thermodynamically favorable alterations facilitated riluzole diffusion, improving its release kinetics from the adhesive matrix.

Frequency sweep analysis demonstrated that the semi-IPN PSA exhibited enhanced viscoelastic performance relative to its linear counterpart, suggesting that its crosslinked architecture provides structural reinforcement that effectively counteracts plasticization-induced destabilization (Chen et al., 2025). With respect to the covalent crosslinking-mediated anti-plasticization effect, plasticization in linear PSA results in molecular chain slippage and reduced mechanical stability. However, the chemically crosslinked nodes within the semi-IPN matrix act as anchor points, that effectively constrain chain mobility, therefore limiting excessive flow and preserving structural integrity under plasticizing conditions (Fig. 3F) (Abel et al., 2021; Clarke et al., 2023). The mechanical performance and thermal stability of the material can be further optimized by modulating the type and concentration of crosslinking agents (Salminen et al., 2021; Zhang et al., 2019). Although PGD disrupts non-covalent interactions such as hydrogen bonding between PSA chains, the presence of a strategically designed stable covalent network maintains the material's elastic recovery, enhancing its mechanical resilience and long-term durability (Yu et al., 2024). This “stiffness–toughness balance” architecture offers distinct advantages for high drug-loading transdermal patches by preserving structural integrity while enabling sustained and controlled drug release (Zhang et al., 2023a). Concurrently, the semi-IPN PSA retains its structural integrity after plasticization, thus maintaining optimal mechanical performance. The dual-optimization strategy embodies a synergistic combination of covalent cross-linking networks that maintain mechanical integrity and PGD-mediated regulation of intermolecular interactions that enhance drug release and permeation, effectively resolving the intrinsic conflict between structural stability and transdermal delivery efficiency in advanced drug delivery systems. The strategy is useful for developing advanced transdermal formulations combining high drug loading with enhanced permeation efficiency.

## 5 Conclusion

This study developed a long-acting riluzole transdermal patch and investigated the PGD release and transdermal absorption through in vitro and in vivo experiments. The results demonstrated that PGD not only enhances the drug release from the patch but also significantly promotes the transdermal absorption of the drug. This study offers valuable insights into the essential role of CPEs in enhancing drug release and transdermal permeation. It serves as a reference point for the strategic selection of CPEs to optimize drug absorption through the skin and provides a research framework for developing extended-release riluzole transdermal patches. The semi-IPN PSA also demonstrates excellent adhesive properties, rendering it suitable for long-acting patches with high drug loading, in turn highlighting its considerable potential for practical applications.

## Funding information

This study was supported by the 10.13039/501100001809National Natural Science Foundation of China (No. 82204306) and Graduate Student Innovation Project of Beihua University (No. [2024]071).

## CRediT authorship contribution statement

**Yanan Liu:** Writing – original draft, Validation, Software, Methodology, Investigation, Funding acquisition, Formal analysis, Data curation, Conceptualization. **Guixue Chen:** Validation, Formal analysis. **Maojian Li:** Visualization, Investigation, Formal analysis. **Man Li:** Investigation, Formal analysis. **Daoxuan Xie:** Validation, Methodology. **Zheng Luo:** Writing – review & editing, Supervision, Resources, Project administration, Funding acquisition, Conceptualization.

## Declaration of competing interest

All authors declared that no conflict of interest existed.

## Data Availability

Data will be made available on request.
